# Twitter-derived neighborhood characteristics associated with obesity and diabetes

**DOI:** 10.1038/s41598-017-16573-1

**Published:** 2017-11-27

**Authors:** Quynh C. Nguyen, Kimberly D. Brunisholz, Weijun Yu, Matt McCullough, Heidi A. Hanson, Michelle L. Litchman, Feifei Li, Yuan Wan, James A. VanDerslice, Ming Wen, Ken R. Smith

**Affiliations:** 1 Department of Department of Epidemiology and Biostatistics, University of Maryland, College Park, School of Public Health, College Park, USA; 2 Institute for Healthcare Delivery Research, Intermountain Healthcare, Salt Lake City, USA; 3 Department of Health, Kinesiology, and Recreation, College of Health, University of Utah, Salt Lake City, USA; 4 Department of Geography, University of Utah, Salt Lake City, USA; 5 Department of Surgery, University of Utah, Salt Lake City, UT USA; 6 College of Nursing, University of Utah, Salt Lake City, USA; 7 School of Computing, University of Utah, Salt Lake City, USA; 8 Utah Population Database, Huntsman Cancer Institute, University of Utah, Salt Lake City, USA; 9 Division of Public Health, Department of Family and Preventive Medicine, University of Utah, Salt Lake City, USA; 10 Department of Sociology, University of Utah, Salt Lake City, USA; 11 Department of Family and Consumer Studies and Population Science & Huntsman Cancer Institute, University of Utah, Salt Lake City, USA

## Abstract

Neighborhood characteristics are increasingly connected with health outcomes. Social processes affect health through the maintenance of social norms, stimulation of new interests, and dispersal of knowledge. We created zip code level indicators of happiness, food, and physical activity culture from geolocated Twitter data to examine the relationship between these neighborhood characteristics and obesity and diabetes diagnoses (Type 1 and Type 2). We collected 422,094 tweets sent from Utah between April 2015 and March 2016. We leveraged administrative and clinical records on 1.86 million individuals aged 20 years and older in Utah in 2015. Individuals living in zip codes with the greatest percentage of happy and physically-active tweets had lower obesity prevalence—accounting for individual age, sex, nonwhite race, Hispanic ethnicity, education, and marital status, as well as zip code population characteristics. More happy tweets and lower caloric density of food tweets in a zip code were associated with lower individual prevalence of diabetes. Results were robust in sibling random effects models that account for family background characteristics shared between siblings. Findings suggest the possible influence of sociocultural factors on individual health. The study demonstrates the utility and cost-effectiveness of utilizing existing big data sources to conduct population health studies.

## Introduction

Where we live, including the social, political, economic, and built environment, impacts health and creates health disparities. Poor access to food^1–6^, abundance of fast food chains^7^, lack of recreational facilities^8,9^, low levels of walkability^10–12^, and high crime rates^2,13^ have been shown to predict higher obesity rates. Environmental exposure to toxicants, noise, and violence can also be detrimental to physical and mental health^14,15^. Conversely, neighborhood resources such as playgrounds for children to play outdoors, access to healthy food, and parks or fitness centers for physical activity engagement can be beneficial to developmental outcomes and chronic disease prevention^1,14,16,17^. Adverse neighborhood conditions often converge in poor and minority neighborhoods^18–21^, thereby increasing health disparities.

Social environments can offer social and emotional support as well that buffers stressful life events^22^. Johns and colleagues found that neighborhoods with higher social cohesion had residents with lower risk of posttraumatic stress disorder^23^. Communities with higher happiness levels have residents with lower rates of obesity, hypertension, and suicide as well as increased life expectancy^24–29^. Health behaviors, such as, healthy food consumption, health screening, smoking, alcohol consumption, drug use and poor sleep patterns have been observed to spread through social networks^30–33^. Social processes and networks can affect health through mechanisms such as the maintenance of norms around health behaviors, the stimulation of new interests, political advocacy for access to resources, emotional support, and dispersal of knowledge about health promotion practices. The social environment can therefore offer opportunities for social control, in regulating unhealthy behaviors and facilitating the social learning of healthy behaviors, but can also promote risky behaviors. Nonetheless, patterns observed in one area may not be applicable to another as characteristics vary by location. One strategy for understanding geographical disparities is through the use of pervasive and publicly available social media data.

The scarcity of data on neighborhood characteristics greatly limits understanding of neighborhood effects on health outcomes. Neighborhood data collection is expensive and time consuming, and then existing data are only available for certain time periods or certain areas^34^. Widespread usage of the Internet and recordings of many transactions (e.g., Yelp reviews, Foursquare check-ins, and reporting of personal opinions and behaviors through social media) has led to the availability of massive amounts of data that enable understanding of previously hidden local area interactions. Social media are also being used to examine public health concerns. Researchers are increasingly utilizing social media and user-generated data to track health behaviors and perform health surveillance (e.g., for outbreak detection)^35–39^. Researchers have utilized social media to track sleep issues^40^, personal health states disclosed by Twitter users^41,42^, and patient-perceived quality of care^43^. Information generated via Twitter can be useful in the examination of beliefs, attitudes, and sentiment towards certain health topics (e.g., vaccines)^44^ as well as health-related activities and health status. Social learning theory posits that learning is a cognitive process that occurs in a social context. Views and activities described via social media can help shape perceived norms, attitudes, beliefs and subsequently, behaviors of people.

### Study Aims and Hypotheses

The aim of this study is to investigate associations between neighborhood characteristics and chronic disease, while accounting for potentially confounding individual and zip code level census characteristics. We create zip code level indicators of community happiness and social modeling of diet and physical activity from tweets (utilizing the location information of where the tweet was sent). We then test these sociocultural contextual factors as predictors of individual level health outcomes. We hypothesize that individuals living in communities that are happier and that model healthy eating and physical activity will have lower prevalence of obesity and diabetes. Data from social media can help uncover prevalent personal attitudes, beliefs, and health-related activities. Combining Twitter data with other sources can allow for the investigation of geographic disparities in health outcomes.

We merge our Twitter data with large-scale administrative data from the state of Utah (driver licenses data with self-reported anthropometrics; birth certificate data with information on birth weight, education, nonwhite race, and Hispanic ethnicity). We examine obesity and diabetes diagnoses utilizing clinical data from the University of Utah Health Sciences Center Enterprise Data Warehouse and Intermountain Healthcare’s Enterprise Data Warehouse. In total, we examine the health outcomes of close to two million individuals in Utah.

## Methods

### Social media data collection and spatial joins

From February 2015–April 2016, we utilized Twitter’s Streaming Application Programming Interface (API) to continuously collect a random 1% sample of publicly available tweets sent from Utah with latitude and longitude coordinates (n = 422,904). The Twitter API is freely available to everyone and this API allows users free access to subsets of public tweets sent from anywhere in the world. Users may request tweets for a certain geographic area, that contain a particular set of keywords, or just random subsets of tweets.

### Processing tweets

Each tweet was divided into tokens using the Stanford Tokenizer^45^, an open access software tool that divides text into tokens, which roughly correspond to “words.” Below, we briefly describe algorithms we utilized to create variables for happiness, and references to food and physical activity from tweets. Please refer to our national trends paper for more details on methodology^46^.

#### Sentiment Analysis

To conduct sentiment analysis, we utilized MAchine Learning for LanguagE Toolkit (MALLET)—a software program that characterizes the sentiment of text^47^. In order to train the software to analyze tweets, which differ from other types of text, we obtained labeled tweets from other research teams^48–50^. MALLET uses these labeled tweets to learn what a human considers a “happy” or “unhappy” tweet. We then run MALLET on our collection of tweets and it assigns to each tweet a probability ranging from 0 to 1 that the tweet is happy. Higher predicted probabilities indicate higher certainty that the tweet is happy.

To decide on a cut point for the MALLET predicted probability at which we would classify tweets as “happy,” two coauthors manually labeled a random 1200 tweets. We computed accuracy levels at different cut points of MALLET predicted probabilities. Using higher cut points improves the accuracy against human annotations but also reduces the calculated prevalence of tweets deemed as happy. A MALLET probability of 0.80 achieves the highest level of accuracy while still maintaining a prevalence of happy tweets of 19% (which approximates the prevalence obtained by human annotations).

#### Food tweets

We compiled a list of over 1,430 popular food words from the U.S. Department of Agriculture’s National Nutrient Database^51^. At the time of download, the USDA database had over 8000 foods listed. We reduced the numbers of foods by eliminating second-level information, which is often not included in tweets. For example, we did not distinguish between fruit juice that was canned, frozen or from concentrate. We also added food dishes not food on the USDA list such as tiramisu by examining food lists from previous Twitter studies^52^ and food lists created by popular press^53^. Each food item was associated with a measure of caloric density, operationalized as calories per 100 grams. Fruits, vegetables, nuts, and lean proteins (e.g., fish, chicken, and turkey) were labeled as “healthy foods” (340 food terms in total). Fried versions of foods (e.g., fried chicken, French fries) were not considered healthy foods. Our food list also contained popular national fast food restaurants, such as McDonald’s and Kentucky Fried Chicken (captured via 154 food terms including popular variations of restaurant names) to enable quantification of fast food references.

To analyze food culture, each tweet was examined for words or phrases matching those on our list. Each food item on our list was described by one or two words. Our text matching algorithm first searched over a tweet for matches to two-word foods (e.g., orange chicken). It then searched over the remaining words for matches to one-word food terms (e.g., taco). We computed caloric density by summing up all the foods mentioned in the tweet.

#### Physical activity tweets

We created a list of physical activities using published lists of physical activity terms gathered from physical activity questionnaires, compendia of physical activities, and popularly available fitness programs^54,55^. Our physical activity list had 376 different activities that incorporate gym-related exercise (e.g., treadmill, weight lifting), sports (e.g., baseball), recreation (e.g., hiking, scuba diving) and household chores (e.g., gardening). We excluded popular phrases that generally do not relate to physical activity such as “*walk away*” and “*running late*.” Using Metabolic Equivalents (METs) associated with physical activities, we quantified the exercise intensity of each physical activity mention, scaled for a duration of 30 minutes and for a 155 pound individual, which approximates the weight of an average American adult^56–58^.

**Figure 1:**
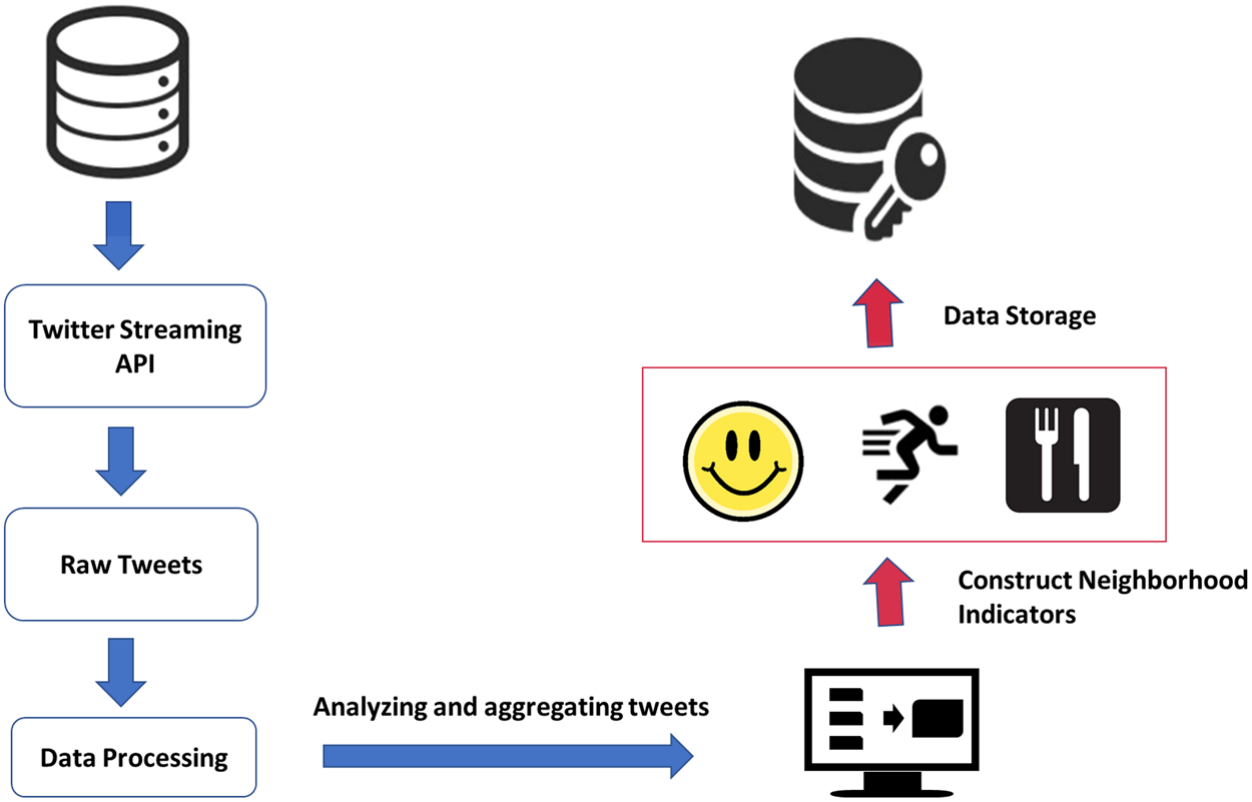
Schematic diagram of data collection and processing of Twitter data. Twitter data was collected using Twitter’s Streaming API. Each tweet was processed to extract its sentiment as well as its food and physical activity mentions. The location information of where the tweet was sent was used to assign the tweet to its corresponding zip code. We created neighborhood indicators by averaging for each zip code—the sentiment of tweets, the frequency and type of food tweets, and the frequency of physical activity tweets. Blue arrow point to steps in processing individual tweets while red arrows point to steps in processing aggregated tweets at regional levels.

Machine-labeled and manually-labeled tweets had a high level of accuracy: 78% for happiness, 83% for food and 85% for physical activity for dichotomized labels, with the following F-scores: 0.86 (food), 0.90 (exercise), and 0.54 (happiness). These variables (i.e. happiness, any food references, caloric density of foot tweets, healthy food references, physical activity references) were then aggregated and summarized at the zip code level to create zip code indicators of the social environment. Figure 1 provides schematic of our Twitter data collection and processing steps.

#### Individual-level health data

Cohort (Utah adults): Our analytic sample consisted of adults aged 20 years and older in 2015 living in Salt Lake City. We merged our neighborhood data with individual outcome data from the Utah Population Database (UPDB). The UPDB is the only database of its kind in the United States and one of the few in the world providing population-wide data for public health studies. Most families in Utah are represented in the UPBD. The central component of UPDB is the extensive set of Utah family histories with links to demographic and medical data. The UPDB contains 19 million records for over 7.3 million individuals with links to demographic (from state administrative records) and medical data (from participating hospitals and clinics). The UPBD is a dynamic database that is updated annually by data contributors. New individuals are added to the UPDB by merging genealogies with birth data. Multiple records for each individual are linked by a unique person id. The UPDB can only be utilized for biomedical and health-related research. Confidentiality and privacy of individuals is strictly protected. The UPDB is provided by the Pedigree and Population Resource (PPR) under the direction of Dr. Ken Robert Smith.

Obesity: Obesity was defined dichotomously as body mass index (BMI) (kg/m^2^) ≥ 30 from Intermountain Healthcare’s Enterprise Data Warehouse. If clinical BMI measurements were missing (42%) because the individual did not seek medical care with Intermountain, BMI data was supplemented by self-reported height and weight extracted from Utah driver licenses. Utah law requires individuals to renew their driver license every five years and to renew in person every ten years. BMI data were drawn from the most recent driver license data available, with dates that ranged from 2011–2017. Using these two data sources in combination (clinical BMI measurements, Utah driver licenses), only 1% of individuals’ obesity status were missing.

Diabetes was assessed via diagnoses by a health care provider using ICD-9 (‘250.00’, ‘250.01’, ‘250.02’, ‘250.03’, ‘250.70’, ‘250.71’, ‘250.72’, ‘250.73’, ‘250.40’, ‘250.41’, ‘250.42’, ‘250.43’, ‘250.50’, ‘250.51’, ‘250.52’, ‘250.53’, ‘250.60’, ‘250.61’, ‘250.62’, ‘250.63’,) and ICD-10 codes (E11.40’, ‘E10.9’, ‘E10.29’, ‘E11.51’, ‘E11.29’, ‘E11.36’, ‘E10.36’, ‘E10.39’, ‘E10.51’, ‘E10.40’, ‘E10.65’, ‘E11.21’, ‘E11.319’, ‘E10.319’, ‘E11.65’, ‘E11.311’, ‘E11.39’, ‘E10.21’, ‘E10.311’, ‘E11.9’). In addition, if available, lab values for glycated hemoglobin (HbA1c (%)) and fasting plasma glucose values (mg/dL) were obtained. Sources of the clinic data included the University of Utah Health Sciences Center Enterprise Data Warehouse (UUHSC EDW), Utah’s Department of Health inpatient and ambulatory surgery data, and Intermountain Enterprise Data Warehouse. Patients were linked across files and sources via a Master Patient Index.

#### Covariates

We included individual socio-demographic characteristics along with health/disease predispositions to adjust for potential confounding of the relationship between neighborhood environments and adult obesity and diabetes. As a clarification, in the United States, race and Hispanic ethnicity are treated as different social constructs following federal standards established by the 1997 Revisions to the Standards for the Classification of Federal Data on Race and Ethnicity^59^. A person who is of Hispanic origin can be white or nonwhite. In our analytic sample, most individuals who report Hispanic ethnicity are white (84%). The remainder of Hispanic individuals report nonwhite race. In the analyses, we control for nonwhite race and Hispanic ethnicity. In total, the following individual-level covariates were included: age (years), sex (male/female), marital status (married vs. divorced/single), race (white/nonwhite), Hispanic ethnicity (yes/no), and education (less than high school, high school, some college, college or greater). We utilized education information appearing on the Utah birth certificates of their children (if any).

Indicator variables were created for missing data on individual level covariates. *Zip code characteristics*. We utilized zip code boundaries as a working definition of neighborhoods. To assess whether our social environment variables were associated with individual health outcomes above and beyond other zip code level characteristics, we obtained five-year estimates (2011–2015) from the American Community Survey for the following zip code level characteristics: population density, percent of the population 65 years and older, percent Hispanic, percent black, and median household income. The study was approved by the University of Utah’s Institutional Review Board and all methods were performed in accordance with the relevant guidelines and regulations. The study utilized publically available big data sources as well as de-identified data from the Utah Population Database and Intermountain Healthcare Data Warehouse (and thus informed consent was not relevant for the study).

### Analytic Approach

#### Mapping

Each geolocated tweet we collected was assigned its corresponding zip code using Python software (version 2.7.12). We created maps using ArcGIS Desktop Version 10.5 (ESRI, Redlands CA, http://www.esri.com/arcgis/about-arcgis) and the 2016 U.S. Census TIGER/Line Shapefiles (https://www.census.gov/geo/maps-data/data/tiger-line.html)^60^. We utilized natural breaks in the data to display spatial patterns.

#### Regression modeling

To examine the relationship between neighborhood characteristics and individual-level health outcomes, we merged administrative and medical records from the Utah Population Database to zip code summaries of social environment characteristics derived from Twitter data and census data. Zip code characteristics were categorized into tertiles and the lowest tertile was utilized as the referent level. We hypothesize that individuals living in areas with more happy tweets and physical activity tweets would have lower obesity and diabetes prevalence. Additionally, we hypothesize that individuals living in areas with higher caloric food tweets would have worse health outcomes. We implemented log Poisson regression models to estimate associations between neighborhood characteristics and chronic disease, accounting for individual sociodemographic characteristics. Reported prevalence ratios represent comparisons between individuals in the 3^rd^ tertile (vs. 1^st^ tertile) and 2^nd^ tertile (vs. 1^st^ tertile) for zip code characteristics. Because our analyses are cross-sectional, the results of the log Poisson regression models are prevalence ratios (rather than risk ratios as would be the case of longitudinal data). Higher prevalence ratios indicate that individuals living in zip codes in the 3rd and 2nd tertile have higher chronic disease prevalence than those in the 1st tertile. Lower prevalence ratios indicate that individuals living in zip codes in the 3rd and 2nd tertile have lower chronic disease prevalence than those in the 1st tertile. We estimated prevalence ratios rather than odds ratios because prevalence ratios are arguably more interpretable; this is because people are more likely to think in terms of probabilities such as 10% and 33% rather than their corresponding odds (1:9 and 1:2 odds)^61^. Additionally, odds ratios can overestimate prevalence ratios for common outcomes^62^. Adjusted linear regression was utilized for continuous outcome variables such as body mass index (kg/m^2^), fasting plasma glucose (mg/dL) and glycated hemoglobin (HbA1c; %). Positive values for linear regression results indicate higher BMI, fasting glucose, and HbA1c, while negative values indicate lower BMI, fasting glucose, and HbA1c values. In sensitivity analyses, we utilized sibling random effects models to control for family and background characteristics shared among siblings. For sibling random effects models, we examined how siblings living in different neighborhood contexts may have different health outcomes.

Missingness on variables varied from less than 1% (obesity) to 39% (education). In sensitivity analyses, multivariate normal imputation was utilized to fill in missing values with predicted values based on information from non-missing covariates (using the mi impute mvn command in Stata). Additionally, we stimulated extreme values for missing data and examined impacts on estimates. Standard errors adjusted for clustering of zip code values within a county. Statistical analyses were implemented with Stata MP13 (StataCorp LP, College Station, TX).

### Data availability

Twitter-derived summaries of happiness, food and physical activity culture at the state, county, zip code and census tract levels can be downloaded here: https://hashtaghealth.github.io/geoportal/start.html.

## Results

**Figure 2:**
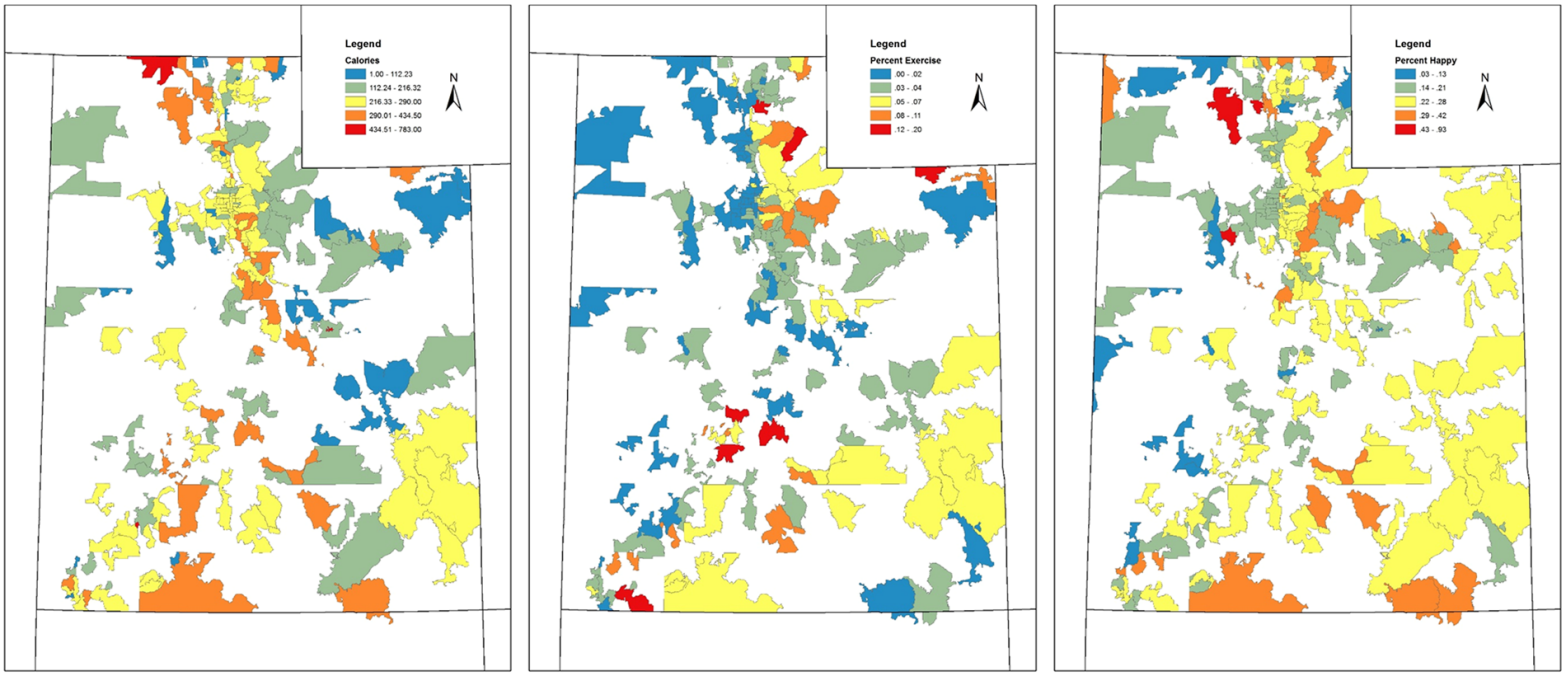
Geographic distribution of Twitter-derived zip code characteristics, Utah. Zip code summaries of caloric density of tweets, percent of tweets about physical activity, and percent of tweets that are happy. Choropleth maps were created using ArcGIS Desktop Version 10.5 (ESRI, Redlands CA, http://www.esri.com/arcgis/about-arcgis) and the 2016 U.S. Census TIGER/Line Shapefiles (https://www.census.gov/geo/maps-data/data/tiger-line.html).

Table 1 presents descriptive statistics for zip code level and individual level characteristics. Across 216 zip codes in Utah, the average prevalence of happy tweets was 22%. On average, 3.1% of tweets mentioned physical activity and 3.8% were related to food. The average caloric density of food tweets (per 100 grams) was 232 calories. Figure 2 displays the geographic distribution of zip code summaries of Twitter-derived variables. At the zip code level, the percentage of happy and physical activity tweets were weakly correlated, *r* = 0.35. All other correlations had absolute values less than *r* = 0.11. Zip codes along the Utah - Idaho border in Box Elder County show a pattern of higher calories, lower exercise and lower happiness percentages. Summit County and Wasatch County are well known for their outdoor recreational activities and lifestyle, and these zip codes just east of the Salt Lake Valley are moderately high in exercise and happiness and low in calories. Larger regional patterns also show that zip codes in the Northwest corner of Utah are lower in exercise, whereas zip codes in the Northeast corner near Vernal and Duchesne County have a cluster of low calories. The South and Southeastern areas of Utah have relatively high percentages of happiness and they are classified as rural and frontier areas with many opportunities for recreation, including hiking and camping.

**Table 1:** Descriptive characteristics for neighborhood and individual characteristics.

	N	Mean (SD)
*Zip code level Twitter characteristics*		
% of tweets that are happy	216	22.06 (11.13)
% of tweets about physical activity	183	3.06 (3.18)
Caloric density of food tweets	216	231.89 (94.94)
*Individual-level characteristics*		
Age (years)	1,968,451	46.23 (17.37)
% Female	1,964,485	49.55 (50.00)
% Married	1,786,137	61.82 (48.58)
% Nonwhite	1,832,286	4.73 (21.23)
% White	1,832,286	95.27 (21.23)
% Hispanic ethnicity	1,703,412	11.05 (31.35)
% Less than high school	1,208,101	9.11 (28.77)
% High school	1,208,101	30.00 (45.83)
% Some college	1,208,101	31.90 (46.61)
% College or greater	1,208,101	28.99 (45.37)
% Obese	1,952,993	28.75 (45.26)
% Diabetes	1,968,451	5.22 (22.24)
Body mass index (kg/m^2^)	1,952,993	27.67 (6.52)
Fasting glucose (mg/dL)	137,589	94.91 (32.15)
Hemoglobin A1c (%)	382,100	6.06 (2.17)

Data sources: 422,904 geolocated tweets from Utah aggregated to the zip code level; Utah Population Database, University of Utah Health Science Center Data Warehouse; Intermountain Healthcare Data Warehouse.

Our analytic sample was restricted to adults 20 years and older in Utah; the mean age was 46 years old (Table 1). The sample is equally divided by sex. About 62% were married and over 95% of individuals were white and 11% were of Hispanic ethnicity (of any race). About 29% of individuals had a college degree or greater. Obesity prevalence was about 29% and diabetes prevalence was about 5.2%.

We find that individuals living in zip codes with the highest percentages of happy tweets (PR = 0.88 (95% CI: 0.81, 0.96) and physical activity tweets (PR = 0.91 (95% CI: 0.85, 0.97) had 9–12% lower obesity prevalence (Table 2). Zip codes with the highest percentages of happy tweets and physical activity tweets also saw a reduction in overweight status (eTable 1). More happy tweets in a zip code (PR = 0.91 (95% CI: 0.83, 0.99) was also associated with lower individual diabetes prevalence (Table 2). Higher caloric density of food tweets for a zip code was related to higher diabetes prevalence. Individuals living in zip codes with the highest percentages of healthy food tweets had lower obesity and diabetes prevalence (eTable 2). The abundance of fast food tweets in a zip code was not related to individual obesity or diabetes. Analyses utilizing continuous BMI and fasting glucose (mg/dL) also suggested a relationship between Twitter-derived positive social environment characteristics and lower chronic disease (Table 2). Results were attenuated and non-significant for glycated hemoglobin (HbA1c (%)).

**Table 2:** Twitter-derived predictors of adult obesity and diabetes (Type 1 and Type 2)^a^.

	Log Poisson Regression for dichotomous outcomes		Linear regression for continuous outcomes		
	Obese	Diabetes	Body Mass Index (kg/m^2^)	Fasting glucose (mg/dL)	HbA1c (%)
*Zip code level Twitter predictors*	Prevalence Ratio (95% CI)^b^	Prevalence Ratio (95% CI)^b^	Beta (95% CI)^b^	Beta (95% CI)^b^	Beta (95% CI)^b^
Happy tweets					
3rd tertile (highest)	0.88 (0.81, 0.96)	0.91 (0.83, 0.99)	−0.57 (−0.94, −0.21)	−1.44 (−2.43, −0.44)	−0.01 (−0.08, 0.07)
2nd tertile	0.94 (0.90, 0.98)	0.97 (0.95, 0.99)	−0.34 (−0.56, −0.12)	−0.86 (−1.44, −0.28)	0.01 (−0.09, 0.11)
Physical activity tweets					
3rd tertile (highest)	0.91 (0.85, 0.97)	0.96 (0.88, 1.05)	−0.39 (−0.71, −0.07)	−0.87 (−2.30, 0.56)	−0.08 (−0.19, 0.03)
2nd tertile	0.96 (0.93, 1.00)	1.00 (0.95, 1.05)	−0.16 (−0.33, 0.01)	−0.70 (−1.18, −0.21)	0.02 (−0.06, 0.10)
Caloric density of food tweets					
3rd tertile (highest)	1.03 (0.98, 1.08)	1.08 (1.01, 1.16)	0.13 (−0.07, 0.34)	0.80 (0.10, 1.49)	0.02 (−0.04, 0.08)
2nd tertile	1.04 (1.00, 1.07)	1.13 (1.06, 1.20)	0.17 (0.03, 0.31)	0.76 (0.11, 1.41)	0.02 (−0.02, 0.06)
N	1,855,768	1,866,509	1,855,768	131,015	362,035

^a^Data source for health outcome: Utah Population Database and Intermountain Healthcare Enterprise Data Warehouse on Utah adults 20 years and older. ^b^Adjusted regression models were run for each outcome separately. For dichotomous outcomes such as obesity and diabetes (0 = no; 1 = yes), log Poisson models were utilized. For continuous variables like body mass index, linear regression was used. Models controlled for age, sex, nonwhite race, Hispanic ethnicity, education, marital status as well as the following zip code area characteristics: population density, percent of the population 65 years and older, percent Hispanic, percent black, and median household income. Indicator variables were created for missing data on covariates. Twitter-derived characteristics were categorized into tertiles, with the lowest tertile serving as the referent group. Standard errors adjusted for clustering of values at the county level.

Variables with the most missing data included education level and Hispanic ethnicity. Using data imputation and alternatively, extreme value substitution for missing values resulted in qualitatively very similar estimates (eTable 3). We additionally adjusted for birth weight, a predisposing factor for obesity and diabetes^63,64^. Results were qualitatively very similar (eTable 4), but the sample size was smaller because analyses were limited to adults who were born in Utah and hence a birth certificate was available with birth weight.

Sibling random effects models that are able to account for the full set of family background characteristics shared between siblings additionally suggested relationships between social environment indicators and obesity and diabetes. In sibling random effects models, more happy tweets and physical activity tweets in a zip code were related to 7–8% reduction in obesity prevalence (Table 3).

**Table 3:** Sibling random effects model results: Twitter predictors of individual health outcomes.

	Log Poisson Regression		Linear Regression
	Obese	Diabetes	Body Mass Index (kg/m^2^)
*Built environment characteristics*	Prevalence Ratio (95% CI)^b^	Prevalence Ratio (95% CI)^b^	Beta (95% CI)^b^
Happy tweets			
3rd tertile (highest)	0.92 (0.91, 0.93)	0.93 (0.90, 0.95)	−0.40 (−0.44, −0.36)
2nd tertile	0.96 (0.95, 0.96)	0.98 (0.96, 1.00)	−0.24 (−0.27, −0.20)
Physical activity tweets			
3rd tertile (highest)	0.93 (0.92, 0.94)	0.99 (0.96, 1.02)	−0.29 (−0.34, −0.25)
2nd tertile	0.96 (0.95, 0.97)	1.00 (0.98, 1.03)	−0.15 (−0.19, −0.12)
Caloric density of food tweets			
3rd tertile (highest)	1.02 (1.01, 1.03)	1.06 (1.03, 1.08)	0.07 (0.04, 0.11)
2nd tertile	1.03 (1.02, 1.04)	1.10 (1.07, 1.13)	0.16 (0.13, 0.19)
N	944,309	946,324	944,309

^a^Data source for health outcome: Utah Population Database and Intermountain Healthcare Enterprise Data Warehouse on Utah adults 20 years and older. ^b^Adjusted regression models were run for each outcome separately. For dichotomous outcomes such as obesity and diabetes (0 = no; 1 = yes), log Poisson models were utilized. For continuous variables like body mass index, linear regression was used. Models controlled for age, sex, nonwhite race, Hispanic ethnicity, education, and marital status as well as the following zip code area characteristics: population density, percent of the population 65 years and older, percent Hispanic, percent black, and median household income. Twitter-derived characteristics were categorized into tertiles, with the lowest tertile serving as the referent group.

## Discussion

As demonstrated by a vast literature, environmental conditions (social, economic, physical characteristics of places) are important determinants of health. The goal of this study was to test associations between social environment factors and individual health outcomes. This study utilized unique data sources—Twitter data as well as massive administrative and clinical data from the state of Utah. The unique nature of our database also allowed us to implement rarely applied sibling random effects models that further account for shared family and background characteristics. We found that individuals living in zip codes with more happy tweets, physical activity tweets, and food tweets with lower caloric density had lower chronic disease prevalence. Our results were robust to a variety of different model specifications. Findings provide initial evidence for targeted population health interventions to improve the health of specific communities to decrease obesity and diabetes risk.

### Studying finding in context

Social media is being leveraged for a variety of public health research activities including identifying individuals with mental health needs^65^, sleep disorders^40^ and providing timely information on infectious disease outbreaks and spread^66^. Our study, which found associations between higher percentage of physical activity tweets, align with the results of a study conducted by Chunara (2013) who found that locations in the United States with more Facebook likes for physical activity had lower obesity rates^67^. Additionally, De Choudhury and colleagues (2016) found places designated as food deserts which lack access to healthy foods, had more Instagram posts of foods that are higher in fat, sugar, and cholesterol^68^. Previous studies have also linked happiness to lower morbidity and mortality^69^.

### Study strengths and limitations

This study is innovative because it demonstrates the utility and cost-effectiveness of using “organic data” not produced for the primary purpose of research. Besides census data, there are few national neighborhood datasets, especially on the social environment, and thus we leverage Twitter data for the purpose of constructing sociocultural characteristics of neighborhoods. Health information on the population of Utah is derived from sources that collect these data for their everyday functioning, which enables our study to include more individuals than would be typically practical to collect for any single study. Lastly, the study furthers our understanding of the potential influence of the social environment on health.

However, this study is subject to several limitations. The study capitalized on unique data sources for individual characteristics and health outcomes that were available on residents of Utah. Utah has the youngest population, highest birth rate, and largest average household size in the U.S^70^. Additionally, the racial and ethnic composition of Utah is not as diverse as the United States as a whole, but diversity has increased substantially in recent decades. In 2013, 13.3% of Utah’s population was Hispanic and 8.3% were foreign-born, compared to 16.9% and 12.9% for the entire United States population respectively^71^. Finally, Utah has lower chronic disease prevalence than most of the country; it is ranked number one and eleven in the United States for the lowest incidence of diabetes and obesity respectively^72,73^. Thus, study results may not be generalizable to locations outside of Utah. In future work, we will expand our analysis to the general U.S. population. Using for instance, residential location information in the National Health Interview Survey (NHIS) to examine national patterns in the relationship between social environment characteristics derived from Twitter and individual health outcomes.

We aggregated data sources from health care institutions who deliver the majority of care in the state of Utah; University of Utah Health Sciences Center and Intermountain Healthcare currently provide about 85% of the care in the state of Utah. However, we may still be under- or over-estimating diagnoses of obesity and diabetes. The diabetes registry did not distinguish between Type 1 and Type 2 diabetes mellitus but our analytic sample was restricted to adults.

Another limitation was that analyses were cross-sectional, and thus the study was unable to evaluate longitudinal or temporal trends. The analyses did not take into account residential histories and the length of time individuals lived in their current communities. We found links between social environment characteristics (prevalent happiness, social modeling of health behaviors) and chronic disease risk—however, our study was unable to identify the directionality of associations. Populations that are healthier may also have happier Twitter posts and may be more likely to socially model healthy behaviors.

For a given zip code, tweets sent from that zip code were utilized to construct indicators of happiness, food and physical activity culture. However, tweets could be sent by both residents and visitors. Additionally, users of social media tend to be younger than the general population; in 2016, 36% of individuals aged 18–29 years old used Twitter compared to 21% of individuals 50–64 years and 10% among those 65+ years^74^. Nonetheless, adoption rates of social media have been steadily increasing^75^. Importantly, mobile access to internet results in individuals from all socioeconomic status tweeting^74^. Tweets also include information rarely found in other neighborhood sources. Twitter users are composed of individuals as well as groups of individuals, organizations, companies and news outlets. Thus, compiling such information may allow for a more comprehensive examination of the social environment as well as community issues and needs. Nonetheless, using social media data, like other data relies upon people’s willingness to report. The content of tweets reflects information that people feel comfortable reporting and may not represent the true spectrum of their feelings or their experiences. Additionally, leveraging big data sources for health research is relatively new, requiring further refinement as seen by Google Flu Trends—which had difficulty accurately forecasting influenza^76^. To fully capitalize on emerging big data sources, further assessment of potential biases and continued development new methods are needed.

## Conclusions

In this study we investigated whether individuals living in social environments that have higher happiness and more social modeling of healthy behaviors have lower rates of obesity and diabetes. We leverage population-wide administrative and clinical data from large health care providers in the state of Utah to examine potential neighborhood influences on individual health outcomes. We constructed neighborhood summaries from Twitter posts. Individuals living in zip codes with high percentages of happy and physically-active tweets had lower obesity prevalence. More happy tweets and lower caloric density of food tweets in a zip code were also linked with lower diabetes. Findings suggest the possible influence of sociocultural factors on individual health. The study demonstrates the utility and cost-effectiveness of utilizing existing big data sources to conduct population health studies.

### Human participant protection

The University of Utah Institutional Review Board approved the study.

## Electronic supplementary material

Supplementary information
