# Supplementary material for: Twitter-derived neighborhood characteristics associated with obesity and diabetes
Source: Sci Rep. 2017 Nov 27;7:16425. doi: 10.1038/s41598-017-16573-1 (PMC5703998; doi:10.1038/s41598-017-16573-1)
Supplement: Supplementary file 1 — Supplementary information [file 41598_2017_16573_MOESM1_ESM.pdf]

Supplemental materials

Twitter-derived neighborhood characteristics associated with obesity and diabetes

AUTHORS: Quynh C. Nguyen*[a], Kimberly D. Brunisholz[b], Weijun Yu[c], Matt McCullough[d], Heidi Hanson[e], Michelle L. Litchman[f]; Feifei Li[g], Yuan Wan[h], James A. VanDerslice[i], Ming Wen[j], Ken R. Smith[k]

AFFLIATIONS: [a] Department of Department of Epidemiology and Biostatistics, University of Maryland, College Park, School of Public Health, United States; [b] Institute for Healthcare Delivery Research, Intermountain Healthcare, Salt Lake City, United States; [c] Department of Health, Kinesiology, and Recreation, College of Health, University of Utah, Salt Lake City, United States; [d] Department of Geography, University of Utah, Salt Lake City, United States; [e] Department of Surgery, University of Utah, Salt Lake City, UT; [f] College of Nursing, University of Utah, Salt Lake City, United States; [g] School of Computing, University of Utah, Salt Lake City, United States; [h] Utah Population Database, Huntsman Cancer Institute, University of Utah, Salt Lake City, United States; [i] Division of Public Health, Department of Family and Preventive Medicine, University of Utah, Salt Lake City, United States; [j] Department of Sociology, University of Utah, Salt Lake City, United States; [k] Department of Family and Consumer Studies and Population Science & Huntsman Cancer Institute, University of Utah, Salt Lake City, United States

CORRESPONDENCE TO: Quynh Nguyen, Department of Epidemiology and Biostatistics, University of Maryland, College Park, School of Public Health, 4200 Valley Drive, College Park, MD 20742, United States

Tel: +1 (301) 405-6425 Email: qtnguyen@umd.edu

eTable 1. Twitter-derived predictors adult overweight status [a]

	Overweight vs. normal weight
Zip code level Twitter predictors	Prevalence Ratio (95% CI)[b]
Happy tweets	
3rd tertile (highest)	0.95 (0.92, 0.98)
2nd tertile	0.97 (0.95, 0.99)
Physical activity tweets	
3rd tertile (highest)	0.96 (0.93, 0.99)
2nd tertile	0.99 (0.97, 1.01)
Caloric density of food tweets	
3rd tertile (highest)	1.01 (0.99, 1.02)
2nd tertile	1.01 (0.99, 1.03)
N	1,283,976

[a]Data source for health outcome: Utah Population Database and Intermountain Healthcare Enterprise Data Warehouse on Utah adults 20 years and older

[b]Adjusted Poisson models controlled for age, sex, nonwhite race, Hispanic ethnicity, education, marital status as well as the following zip code area characteristics: population density, percent of the population 65 years and older, percent Hispanic, percent black, household income. Indicator variables were created for missing data on covariates. Standard errors adjusted for clustering of values at the county level.

eTable 2. Food predictors of adult obesity and diabetes[a]

	Obese	Diabetes
Zip code level Twitter predictors	Prevalence Ratio (95% CI)[b]	Prevalence Ratio (95% CI)[b]
Healthy food tweets[c]		
3rd tertile (highest)	0.91 (0.87, 0.96)	0.91 (0.86, 0.96)
2nd tertile	0.97 (0.93, 1.01)	0.97 (0.91, 1.03)
Fast food tweets[d]		
3rd tertile (highest)	1.00 (0.95, 1.06)	1.02 (0.94, 1.10)
2nd tertile	1.00 (0.97, 1.02)	0.98 (0.94, 1.02)
N	1,855,768	1,866,509

[a]Data source for health outcome: Utah Population Database and Intermountain Healthcare Enterprise Data Warehouse on Utah adults 20 years and older.

[b]Adjusted Poisson models were run for each outcome separately. Models controlled for age, sex, nonwhite race, Hispanic ethnicity, education, marital status as well as the following zip code area characteristics: population density, percent of the population 65 years and older, percent Hispanic, percent black, household income. Indicator variables were created for missing data on covariates. Standard errors adjusted for clustering of values at the county level.

[c]Healthy foods: Fruits, vegetables, nuts, and lean proteins (e.g., fish, chicken, and turkey). Fried foods (e.g., French fries) were not considered healthy foods.

[d]Fast food: Popular national fast food restaurants, such as McDonald’s and Kentucky Fried Chicken.

eTable 3. Sensitivity analyses with missing data

	Data imputation[a]		Substitute with extreme values[b]	
	Obese	Diabetes	Obese	Diabetes
Zip code level Twitter predictors	Prevalence Ratio (95% CI)[c]	Prevalence Ratio (95% CI)[b]	Prevalence Ratio (95% CI)[c]	Prevalence Ratio (95% CI)[c]
Happy tweets				
3rd tertile (highest)	0.87 (0.80, 0.95)	0.89 (0.80, 0.99)	0.88 (0.81, 0.96)	0.91 (0.83, 0.99)
2nd tertile	0.93 (0.89, 0.97)	0.96 (0.93, 0.99)	0.94 (0.90, 0.98)	0.97 (0.95, 0.99)
Physical activity tweets				
3rd tertile (highest)	0.91 (0.85, 0.97)	0.97 (0.88, 1.06)	0.91 (0.85, 0.97)	0.97 (0.89, 1.06)
2nd tertile	0.96 (0.93, 1.00)	1.01 (0.96, 1.07)	0.96 (0.93, 1.00)	1.01 (0.96, 1.06)
Caloric density of food tweets				
3rd tertile (highest)	1.04 (0.99, 1.09)	1.12 (1.04, 1.21)	1.03 (0.98, 1.08)	1.09 (1.01, 1.17)
2nd tertile	1.05 (1.02, 1.09)	1.17 (1.08, 1.27)	1.04 (1.01, 1.07)	1.14 (1.07, 1.23)
N	1,877,433	1,877,433	1,855,768	1,855,768

[a]Stochastic imputation was utilized to impute missing data values.

[b] Most of the missing data was for Hispanic ethnicity and education level—variables which served as controls. Examining patterns in missing data, we find that individuals with missing data on ethnicity or education level had lower BMI and diabetes prevalence. In our analytic sample, Hispanic individuals and individuals with higher education have lower obesity and diabetes prevalence. Thus, in sensitivity analyses, for those missing education information, we simulated the situation that they had a college degree or greater and for those missing Hispanic ethnicity information, we simulated that they were Hispanic.

[c]Adjusted regression models were run for each outcome separately. For dichotomous outcomes such as obesity and diabetes (0=no; 1=yes), log Poisson models were utilized. For continuous variables like body mass index, linear regression was used. Models controlled for age, sex, nonwhite race, Hispanic ethnicity, education, marital status as well as the following zip code area characteristics: population density, percent of the population 65 years and older, percent Hispanic, percent black, household income. Indicator variables were created for missing data on covariates. Standard errors adjusted for clustering of values at the county level.

eTable 4. Twitter-derived predictors of individual-level health outcomes[a] controlling for weight at birth

	Obese	Diabetes
Zip code level Twitter predictors	Prevalence Ratio (95% CI)[b]	Prevalence Ratio (95% CI)[b]
Happy tweets		
3rd tertile (highest)	0.91 (0.86, 0.96)	0.94 (0.86, 1.02)
2nd tertile	0.95 (0.93, 0.98)	0.97 (0.94, 1.01)
Caloric density of food tweets		
3rd tertile (highest)	1.02 (0.99, 1.05)	1.06 (0.99, 1.14)
2nd tertile	1.04 (1.02, 1.06)	1.08 (1.02, 1.15)
Physical activity tweets		
3rd tertile (highest)	0.92 (0.88, 0.97)	0.97 (0.91, 1.04)
2nd tertile	0.96 (0.94, 0.99)	1.00 (0.95, 1.05)
N	852,741	854,518

[a]Data source for health outcome: Utah Population Database and Intermountain Healthcare Enterprise Data Warehouse on Utah adults 20 years and older.
[b]Adjusted Poisson models were run for each outcome separately. Models controlled for age, sex, nonwhite race, Hispanic ethnicity, education, marital status, and birth weight as well as the following zip code area characteristics: population density, percent of the population 65 years and older, percent Hispanic, percent black, household income. Standard errors adjusted for clustering of values at the county level.
